# *N*^4^-Hydroxycytidine/molnupiravir inhibits RNA virus-induced encephalitis by producing less fit mutated viruses

**DOI:** 10.1371/journal.ppat.1012574

**Published:** 2024-09-30

**Authors:** Durbadal Ojha, Collin S. Hill, Shuntai Zhou, Alyssa Evans, Jacqueline M. Leung, Christine A. Schneider, Franck Amblard, Tyson A. Woods, Raymond F. Schinazi, Ralph S. Baric, Karin E. Peterson, Ronald Swanstrom

**Affiliations:** 1 Laboratory of Neurological Infections and Immunity, Rocky Mountain Laboratories, National Institute of Allergy and Infectious Disease, National Institutes of Health, Hamilton, Montana, United States of America; 2 Lineberger Comprehensive Cancer Center, University of North Carolina at Chapel Hill, Chapel Hill, North Carolina, United States of America; 3 Department of Microbiology and Immunology, University of North Carolina at Chapel Hill, Chapel Hill, North Carolina, United States of America; 4 Research Technologies Branch, Rocky Mountain Laboratories, National Institute of Allergy and Infectious Disease, National Institutes of Health, Hamilton, Montana, United States of America; 5 Center for ViroScience and Cure, Laboratory of Biochemical Pharmacology, Department of Pediatrics, Emory University School of Medicine and Children’s Healthcare of Atlanta, Atlanta, Georgia, United States of America; 6 Department of Epidemiology, University of North Carolina at Chapel Hill, Chapel Hill, North Carolina, United States of America; 7 Department of Biochemistry and Biophysics, University of North Carolina at Chapel Hill, Chapel Hill, North Carolina, United States of America; Georgia State University, UNITED STATES OF AMERICA

## Abstract

A diverse group of RNA viruses have the ability to gain access to the central nervous system (CNS) and cause severe neurological disease. Current treatment for people with this type of infection is generally limited to supportive care. To address the need for reliable antivirals, we utilized a strategy of lethal mutagenesis to limit virus replication. We evaluated ribavirin (RBV), favipiravir (FAV) and *N*^4^-hydroxycytidine (NHC) against La Crosse virus (LACV), which is one of the most common causes of pediatric arboviral encephalitis cases in North America and serves as a model for viral CNS invasion during acute infection. NHC was approximately 3 to 170 times more potent than RBV or FAV in neuronal cells. Oral administration of molnupiravir (MOV), the prodrug of NHC, decreased neurological disease development (assessed as limb paralysis, ataxia and weakness, repeated seizures, or death) by 31% (4 mice survived out of 13) when treatment was started on the day of infection. MOV also reduced disease by 23% when virus was administered intranasally (IN). NHC and MOV produced less fit viruses by incorporating predominantly G to A or C to U mutations. Furthermore, NHC also inhibited virus production of two other orthobunyaviruses, Jamestown Canyon virus and Cache Valley virus. Collectively, these studies indicate that NHC/MOV has therapeutic potential to inhibit viral replication and subsequent neurological disease caused by orthobunyaviruses and potentially as a generalizable strategy for treating acute viral encephalitis.

## Author summary

Mutagenic ribonucleosides can be viewed as broadly acting antivirals against RNA viruses. These viruses have small genomes of essential genes and thus are susceptible to enhanced mutation rates as an antiviral strategy. Here we show that the mutagenic ribonucleoside *N*^4^-hydroxycytidine (NHC) and its prodrug molnupiravir are active against the negative strand bunyavirus La Crosse Virus. Loss of viral infectivity was proportional to the density of mutations induced by NHC. Using a mouse model, we showed that oral administration of molnupiravir attenuated encephalitis caused by La Crosse virus and resulted in reduced virus titers and less fit virus in the brain. This treatment approach should be widely applicable to acute RNA virus diseases, especially those with high morbidity and an absence of other treatment options.

## Introduction

There is a diverse set of RNA viruses that do not circulate in the human population but when introduced from zoonotic sources result in high rates of mortality. Some of these viruses are capable of crossing into the central nervous system (CNS) where they can infect brain cells and induce severe neurological disease including encephalitis or death. This diverse group includes Rabies virus (rhabdovirus), La Crosse virus (peribunyavirus), Nipah virus (paramyxovirus), Eastern and Western Venezuelan equine encephalitis viruses (togaviruses), West Nile virus (flavivirus), Japanese encephalitis virus (flavivirus), Zika virus (flavivirus), tick-borne encephalitis virus (flavivirus), as well as several others [1,2]. Infections in the human population are sporadic and unpredictable. Some of these viruses are arboviruses that spread to humans through an insect vector, while others spread through contact with an infected mammalian host. While these represent diverse viruses in terms of their genome structures and replication strategies, they all share the features of having a small RNA genome tightly packed with essential genes needed for viral replication and host defense evasion.

Two repeatedly successful strategies for developing antivirals are targeting a viral protease as has been done for human immunodeficiency virus (HIV), hepatitis C virus (HCV), and SARS-CoV-2 [3–5], or causing chain termination during viral genome synthesis [6,7], although other viral proteins are now being targeted and in some cases targeting a host protein can be useful. Targeting a specific viral protein function results in an inhibitor that is typically very specific to a group of closely related viruses. Chain-terminating inhibitors can be effective if the viral polymerase will incorporate the analog and the incorporated chain-terminating nucleotide is not removed by a repair or error correction function of the virus. To date, neither of these antiviral drug development strategies has produced a broadly acting inhibitor that could be used on the diverse set of RNA viruses that cause medically important sporadic infections in humans.

Neurotropic RNA viruses do share one vulnerability, their evolutionarily unique RNA dependent RNA polymerase (RdRp). Due to the low fidelity of RdRp, RNA virus replication is error prone, however the introduction of random mutations by RdRp is limited to a rate that is below a level that results in overall harm to the viral population in the form of excess mutations. The antiviral strategy of lethal mutagenesis posits that a quick accumulation of the deleterious mutations in these viruses can go beyond the capacity of natural selection to remove such mutations from the population [8, 9], leading to the loss of fitness and thus favoring host recovery. This strategy was first shown in model systems for a mutagenic deoxyribonucleoside acting against HIV-1 [10] and then a mutagenic ribonucleoside (ribavirin) acting against poliovirus [11]. Mutagenic nucleosides have the ability to pair ambiguously during viral replication, forming either A-U or G-C base pairs but when reversing specificity between the two types of base pairs inducing a transition mutation. Two early compounds (ribavirin and favipiravir) that have been used as therapeutics can give rise to similar mutagenic nucleotides in the cell that can induce mutations *in vitro* [12–16]. However, the levels of drug attained *in vivo* are far below the effective doses needed *in vitro*, making it unclear if these inhibitors can function as effective mutagens in the context of a replicating RNA virus, and their use at high concentrations leads to other effects on the cell [17]. In contrast, the mutagenic nucleoside *N*^4^-hydroxycytidine (NHC) is far more potent *in vitro* where it is possible to show a clear relationship between antiviral activity and mutation load in the viral genome, and its antiviral activity has been shown against a diverse group of RNA viruses [18–22]. The 5’-isopropyl ester prodrug of NHC, molnupiravir (MOV), is active in *in vivo* models for treating respiratory virus infections and is being used to treat SARS-CoV-2 in humans [23–30].

Given that all RNA viruses share the vulnerability of small RNA genomes packed with essential genes, we reasoned that a potent mutagenic nucleoside such as NHC could be active against neurotropic RNA viruses. To test this idea, we selected La Crosse virus (LACV), a negative strand Bunyavirus, to assess the activity of NHC *in vitro* and the utility of its prodrug MOV in a neuropathogenic mouse model. LACV is one of the most common causes of pediatric arboviral encephalitis in North America [2]. We found that both NHC and MOV showed promising therapeutic potential in multiple neuronal cell lines and in two different mouse models by inducing less fit and mutated viruses.

## Material and Methods

### Compounds

For all cell culture experiments, *N*^4^-β-hydroxycytidine (NHC) was obtained through the National Institutes of Health AIDS Reagent Program, Division of AIDS, National Institute of Allergy and Infectious Disease, National Institutes of Health (courtesy of Dr. Raymond Schinazi at Emory University), as a solid that was dissolved in DMSO [31]. For all mouse model experiments, Molnupiravir (MOV) in greater than 98% purity was supplied by the laboratory of Dr. Schinazi at Emory University, Atlanta, Georgia, USA.

### Cells and viruses

Vero cells (African green monkey kidney cells, ATCC) were used for the initial screening study and also for viral titer determination by plaque assays, virus maintenance and virus stock production. LACV (human 1978 strain) was a gift from Richard Bennet (National Institute of Allergy and Infectious Disease) and was used less than 3 passages in Vero cells. LACV was sequenced following the initial passage to confirm the correct sequence of the virus and no contamination. N2a cells (ATCC CCL-131) were grown and maintained in Dulbecco’s modified Eagle medium (DMEM) containing 4,500 mg/L D-glucose, 4 mM L-glutamine and 1mM sodium pyruvate supplemented with 10% fetal bovine serum (FBS). Human (H9) embryonic stem cells-derived hNSCs (Thermo Fisher Scientific) were cultured in KnockOut D-MEM/F-12 media (Thermo Fisher Scientific).

### Antiviral efficacy study by virus infectivity quantification and EC_50_ determination

Cells were seeded in 24-well plates (1 × 10^5^ cells/well) and infected with LACV (0.1 MOI). At 1 hour post infection (hpi), medium was replaced with fresh medium containing 6 different concentrations for each of the drugs. RBV and FAV were tested at concentrations of 1, 3, 10, 30, 100, and 300 μM, while NHC was tested at concentrations of 0.1, 0.3, 1, 3, 10 and 30 μM. DMSO was used as vehicle control. At 24 hpi, the cell supernatant was harvested, centrifuged to eliminate cell debris and stored at −80 °C for sequencing (described below) and plaque assay following the method of Ojha et al. 2021 [32]. Quantified virus titers were plotted to represent antiviral efficacy. The EC_50_ (50% effective concentration representing a 50% reduction in virus titer) was determined by extrapolating a dose-response curve using GraphPad Prism 8 and 9.

### Quantification of LACV viral RNA genome copy number by qRT-PCR

Viral RNA was isolated using the Zymo Direct-zol RNA MiniPrep Kit (Zymo Research) per the manufacturer’s directions. First-strand complementary DNA (cDNA) was generated using iScript Reverse Transcriptase (Bio-Rad). qRT-PCR was performed using PowerUp SYBR Green Master Mix (ThermoFischer) on QuantStudio 6 (Applied Biosystems). Primers used for the reaction were synthesized by Integrated DNA Technologies (IDT) to quantify LACV viral RNA, targeting the M segment of the genome: forward primer 5’-ATTCTACCCGCTGACCATTG-3’, reverse primer 5’-GTGAGAGTGCCATAGCGTTG-3’.

### Primer ID sequencing of viral RNA

We used a Primer ID (PID) library preparation approach with Illumina MiSeq next-generation sequencing (NGS) to examine the mutagenic effects of these drugs on viral RNA allowing deep coverage of the viral population while maintaining a low method error rate (1 in 10,000 nucleotides) [33–35]. We designed cDNA primers targeting a 372 bp region of the RdRp gene within the L segment (referred as L7 in the study), and included a second amplicon covering a 455 bp portion of the M segment (referred as M4 in the study). Each cDNA primer included a block of random nucleotides (11 bases long) as the Primer ID/unique molecular identifier (UMI). Extracted viral RNA was reversed transcribed using SuperScript III (Thermal Fisher) to make cDNA in a single reaction with the two different cDNA primers. After bead purification, the cDNA was amplified by PCR using a mixture of forward primers and a universal reverse primer, followed by a second round of PCR to incorporate Illumina sequencing adapters and barcodes within the amplicons. After gel purification/extraction of the PCR product (Qiagen MinElute Gel Extraction kit), the extracted libraries were quantified using a Qubit fluorimeter and subjected to Agilent TapeStation analysis. For each sequencing run, 24 quantified libraries were pooled for MiSeq 300 base paired-end sequencing at the UNC High Throughput Sequencing Facility. Primers used for the library prep can be found in S1 Table. The initial Primer ID data were processed using the Illumina bcl2fastq pipeline (version 2.20.0), and then template consensus sequences (TCSs) were constructed using the tcs pipeline version 2.5.1 (https://primer-id.org/) [33]. Each type of substitution rate was calculated against the wildtype sequence at the sequenced regions. The sequencing date were submitted to the National Center for Biotechnology Information (NCBI) Sequence Read Archive (SRA), accession number is PRJNA1092655.

### LACV encephalitis (LACV-E) mouse model

All animal experiments were performed following the protocols 2019-023-E and 2022-019-E approved by the National Institute of Allergy and Infectious Disease/National Institutes of Health/Rocky Mountain Laboratories Institutional Animal Care and Use Committee. For this study, a mix of male and female C57BL/6 mice at 23 to 25 days old were used. Mice were divided evenly and infected with LACV at either 1 × 10^3^ pfu by an IP route or 1 x 10^2^ pfu by an IN route. IP-infected mice were started on treatment with MOV at either the date of infection or at 3 dpi; the IN-infected mice were started on treatment on the day of infection. MOV was diluted in 10% polyethylene glycol (PEG), 2.5% Cremophor RH40, and 87.5% sterile water which was used as vehicle control. For oral administration, 300 mg/kg of MOV twice a day was given for up to 10 consecutive days using a straight 24-gauge ball-tip disposable feeding needle (Instech). Mice were examined twice daily for 28–30 days for clinical signs of LACV-induced neurologic disease, which mostly involved limb paralysis, ataxia, or repeated seizures. Mice showing one or more of these clinical symptoms were scored as having clinically significant disease and were subsequently euthanized for tissue removal. Occasionally, LACV-infected mice died from the acute seizures. These mice were also scored as having clinically significant disease and underwent tissue removal when possible.

### Viral load in the CNS and peripheral tissues

Brain tissue and lymph nodes were harvested from mice with clinical symptoms (as described above) or at 5 dpi and stored at -80°C. Half of the sagittal section of the brain tissue was used for plaque assays, and the other half for RNA isolation for qRT-PCR and sequencing. For the plaque assay, brain tissues were homogenized in the presence of DMEM using the Bead Mill 24 (Fisher Scientific) homogenizer at 5,300 rpm for 25 seconds. Homogenized samples were centrifuged at 5000 x *g* for 10 min. Supernatants were then diluted in DMEM containing 10% FBS to 10^−1^ to 10^−7^ dilutions and subjected to a plaque assay [36].

### Measurement of plaque size

We used a digital scanner to scan the plaque assay plates. Digitized images were imported into FIJI/ImageJ v1.52n [37], and pixel calibration values were calculated based on the well diameter and corresponding digitized image. To quantify plaque size (surface area), wells with clearly isolated plaques were selected for segmentation and analysis. The image lookup table for each well was inverted followed by binarization prior to analysis, with a minimum area of 0.39 mm^2^ to eliminate false positives due to slight variations in crystal violet staining. The measurements were imported into Microsoft Excel for data compilation, and graphed and analyzed using GraphPad Prism v9.3.1. Analysis of plaque size was done only when the plaque assays for different virus samples were done in parallel.

### Serial passages of LACV in the presence of NHC

Vero cells were seeded at 5×10^5^ cells/well in a 6-well plate for each passage. For the first passage, Vero cells were infected with LACV at MOIs of 10, 0.1, and 0.001 in 3 ml of medium, and treated with 0.1 μM of NHC or DMSO vehicle control. After 3 dpi, the virus inoculum was harvested from wells where more than 25% of cells were still attached to the well (using light microscopy). The harvested supernatants were centrifuged to clarify dead cells and then used to reinfect Vero cells at dilutions of 1:1,000, 1:100,000, and 1:10,000,000 and treated with 0.1 μM NHC for the first series of 5 passages where at each passage the culture with the highest dilution of virus that still gave rise to incomplete CPE (i.e. 25% of the cells still attached) was saved for the next passage. Second, third, and fourth series of 5 passages were treated with 0.2, 0.4 and 0.8 μM of NHC, respectively. After 20 passages in total, virus suspensions were collected, centrifuged, and frozen at −80°C to perform dose-dependent antiviral activity and plaque assays for virus titers (as described above).

### Statistical analysis

All post-study statistical analyses were conducted using GraphPad Prism v.8.02 and v.9.3.1. All P values are represented as *P<0.05, **P <0.01, ***P<0.001, and ****P<0.0001. Specific analysis information is described in figures legends.

## Results

### Effect of mutagenic nucleoside analogs on production of infectious LACV in multiple cell lines

**Fig 1:**
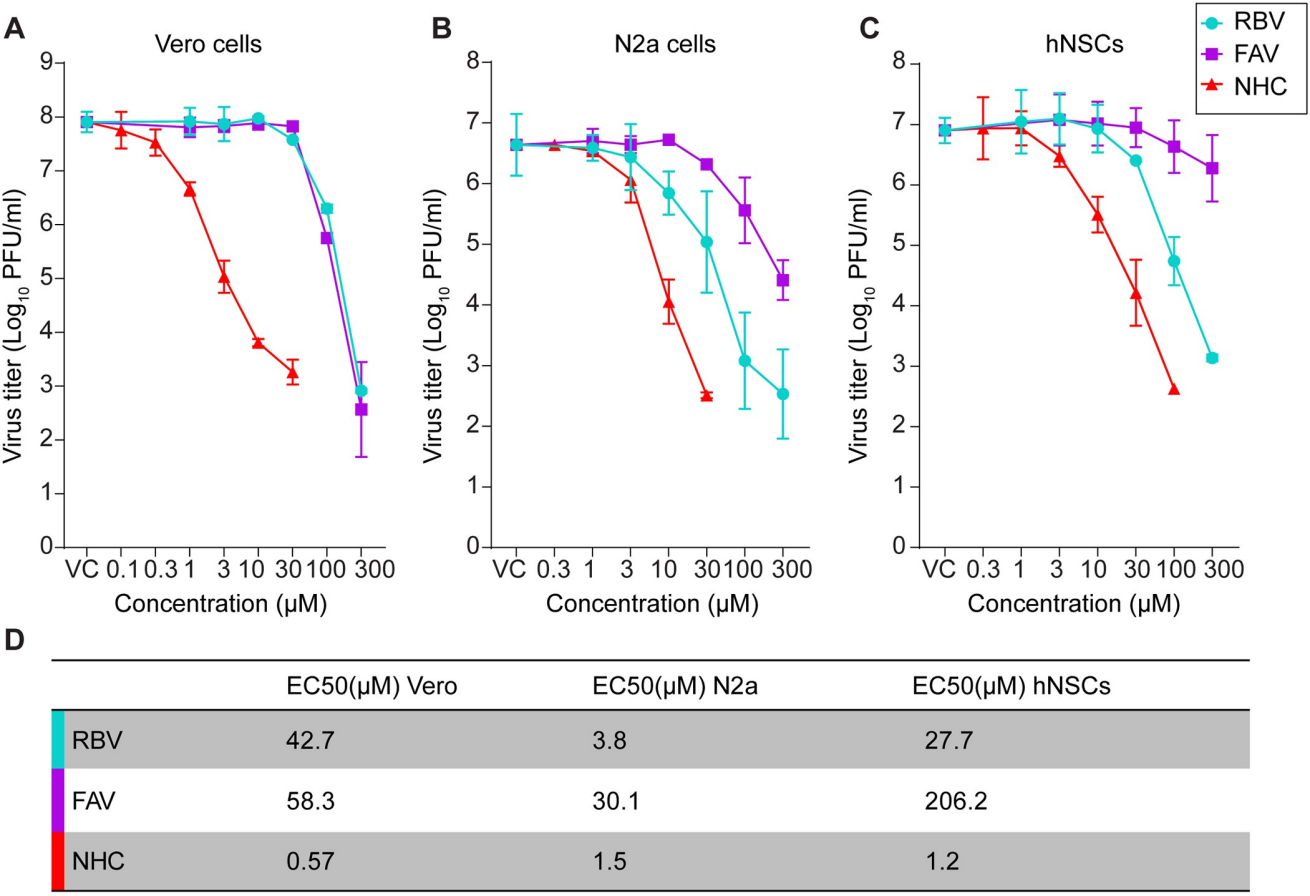
Treatment with NHC inhibits LACV replications more potently than RBV or FAV in multiple cell types. (A-C) Dose-dependent antiviral activity of RBV, FAV and NHC. Cells were treated with different concentrations of either RBV or FAV or NHC or DMSO (vehicle control/VC) immediately after virus adsorption to the cells. At 24 hr post-infection (hpi), virus titer was measured in the cell supernatant by plaque assay. Each line graph represents the mean ± s.d. of infectious viruses present in two individual wells in each of two independent experiments (four wells total) per cell type. (A) Titration of virus produced from drug-treated Vero cells; (B) Titration of virus produced from drug-treated N2a cells; (C) Titration of virus produced from drug-treated hNSC cells; (D) The EC_50_ (median effective concentration) was determined by extrapolating the dose-response curve of either RBV or FAV or NHC in Vero, N2a cells, and hNSCs.

We evaluated ribavirin (RBV), favipiravir (FAV) and *N*^4^-hydroxycytidine (NHC) against LACV infection in Vero cells for inhibition of virus production using six concentrations, ranging between 1 to 300 μM for RBV and FAV, and 0.1 to 30 μM for NHC. A 10-fold lower drug concentration was used for NHC compared to RBV and FAV due to higher cytotoxicity (S2 Table). As expected, all three drugs showed significant reductions in viral titers at the higher concentrations of inhibitor (Fig 1A). The median Effective Concentration (EC_50_) for NHC was 0.57 μM, which was roughly 100 times lower than the EC_50_ of either RBV or FAV, which were 42.7 μM and 58.3 μM, respectively (Fig 1D).

As neurons in the CNS are the primary site of virus replication and tissue damage in humans and in our experimental animal models, we further evaluated each of these nucleoside/base analogs in rodent-derived neuroblastoma Neuro-2a cells (N2a) and human neural progenitor stem cells (hNSCs) (Fig 1B and 1C). In these cells, NHC and RBV markedly inhibited LACV replication, with an EC_50_ of 1.5 μM in N2a cells and 1.2 μM in hNSCs for NHC and an EC_50_ of 3.8 μM in N2a cells and 27.7 μM in hNSCs for RBV (Fig 1D). FAV had a moderate effect on LACV replication in N2a cells and a minimal effect in hNSCs, with EC_50_s of 30.1 and 206.2 μM, respectively (Fig 1D). This is consistent with our recent results showing FAV inhibits LACV replication in Vero cells but not in neuronal cells [32]. Overall, NHC was approximately 3 to 170 times more potent than RBV and FAV, which was consistent with our previous studies that NHC is significantly more active than FAV or RBV against SARS-CoV-2 [22]. Cell biosynthesis of precursors for RNA synthesis results in nucleoside monophosphates thus each of these inhibitors must enter a salvage pathway to become biologically active as a nucleoside triphosphate; differences in metabolic activity of these pathways in different cells could account for the varying EC_50_ values since the effect of mutations on the virus would be expected to be the same irrespective of cell type given an equal concentration of the drug in the total nucleoside triphosphate pool. If the reduced potency of FAV and RBV occurs in spite of the fact that they may also target host functions or the viral RdRp [38–41], this only emphasizes the potency of NHC through a mutagenic mechanism of action.

### Effect of molnupiravir (MOV) on LACV-induced neurological disease in a highly pathogenic mouse model

**Fig 2:**
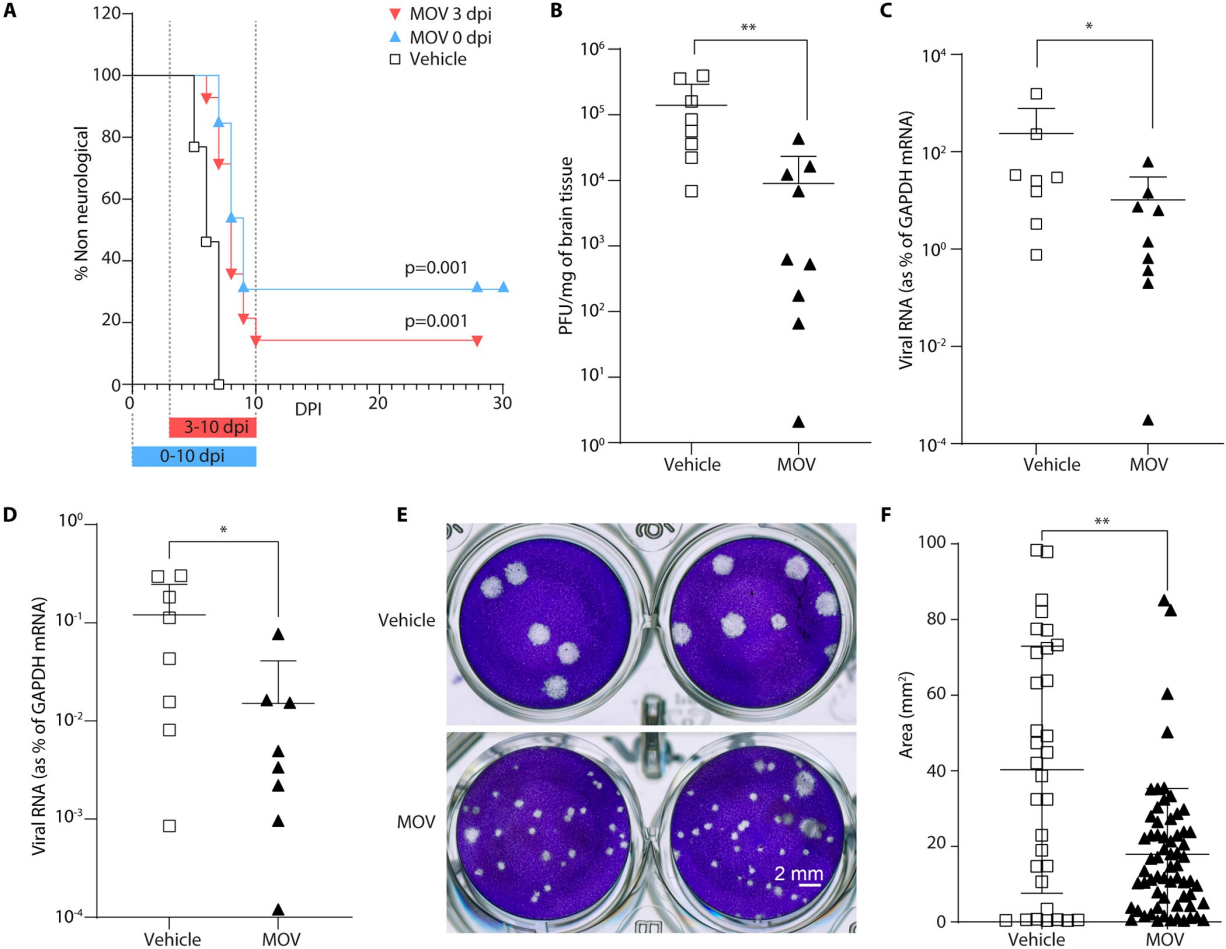
Molnupiravir (MOV) treatment increases survival of LACV-infected mice by inducing less fit and mutated virus in brain tissue. (A) Male and female C57BL/6 mice aged 23 to 24 days were infected IP with LACV (1 x 10^3^ pfu/mouse). Mice were treated orally with MOV (300 mg/kg) or vehicle (n = 13) twice daily, starting at day 0 dpi (2 hr prior to infection) in one group (n = 13) and 3 dpi in another group (n = 14). Kaplar-Meier analysis was used to calculate the difference of clinical disease onset out to day 28–30. (B-F) To assess virus replication and fitness we infected another cohort of mice IP with 1x10^3^ pfu with and without MOV treatment started on the day of infection as above. On day five the mice were sacrificed and brain and lymph node tissues were harvested. (B) Infectious virus was quantified in brain homogenate using a plaque assay (n = 8 for vehicle control and n = 9 for MOV treatment), and (C, D) for viral-RNA by qRT-PCR from (C) brain (n = 8 for vehicle control and n = 9 for MOV treatment) and (D) lymph node (n = 8 for vehicle control and n = 8 for MOV treatment). Each symbol is shown as an individual animal from two different independent experiments. Lines and error bars for each set indicate mean ± s.d. (E) Smaller plaque sizes were observed in plaque assays of virus from brain tissue in the mice receiving MOV treatment compared to mice from the vehicle control group. (F) To quantify the size of plaques (surface area), wells with clearly isolated plaques were digitized and imported into FIJI for segmentation and analysis. Lines and error bars for each set indicate the mean ± s.d. Statistical comparison in (B-D) and (F) were done using the Mann–Whitney U test. For C and D the Ct values for viral RNA and GAPDH were subtracted from each other then used as the exponent of 2 to determine the fold difference, which was multiplied by 100 to give the percent difference, and this number was used in the statistical analysis.

LACV infection of 23–24 day old C57BL/6 mice by intraperitoneal (IP) inoculation (10^3^ pfu/mice) results in lethal viral encephalitis by 5–7 days post infection (dpi). We tested whether MOV could inhibit LACV-induced encephalitis (LACV-E) in mice, by starting treatment on the day of infection or at 3 dpi, with continued treatment to 10 dpi. The choice of a treatment group starting 3 dpi was based on this timepoint showing consistent presence of virus in the CNS by this time after a peripheral inoculation [42]. The prodrug of NHC, MOV, was given to the mice by oral gavage (300 mg/kg) twice each day. The animals were euthanized with the onset of clinical signs of LACV-induced neurologic disease. Both treatment regimens with MOV showed modest efficacy in decreasing the incidence of neurological disease, representing a significant difference from the vehicle-treated mice (Fig 2A). All of the vehicle-treated mice succumbed to viral encephalitis within the first 7 days of infection. When treatment with MOV was started on the day of infection, 31% of the mice survived to 28–30 dpi (Fig 2A). When treatment with MOV was delayed until 3 dpi, only 15% of the mice survived through 28–30 dpi (Fig 2A). Thus, MOV was active as an antiviral *in vivo* providing partial protection in this highly pathogenic model.

### Molnupiravir (MOV) treatment induces less fit and mutated LACV in mouse brains

We carried out an additional mouse infection experiment to examine the effect of MOV treatment on virus load and fitness. In this experiment, we infected mice IP with a dose of 1x10^3^ pfu of LACV per mouse. Treatment was started on day 0 and the animals were euthanized on day 5, the day when the control mice started showing morbidity. To confirm the antiviral activity of MOV *in vivo*, we analyzed brain tissue for virus titer using a plaque assay and for viral RNA using qRT-PCR; we also analyzed the presence of viral RNA in lymph nodes using qRT-PCR. The infectious virus titer (Fig 2B) and viral RNA load (Fig 2C) from the brain tissue and from lymph nodes (Fig 2D) taken at day 5 were significantly decreased in MOV-treated mice compared to vehicle control-treated mice. In both the vehicle control and the MOV-treated animals, the amount of viral RNA was approximately 100-fold lower in the lymph nodes than in brain tissue, emphasizing the neurotropic nature of this virus. Thus, MOV-mediated reduction of LACV-induced neurological disease was associated with a decreased amount of virus in the periphery and in the CNS.

In addition to reduced virus titers in the brain tissue of MOV-treated mice, we noted that the viral plaques from virus isolated from the treated animals appeared to be on average smaller than the plaques from the virus isolated from control animals (Fig 2E). This could be the case if the mutated genomes were still infectious but carried deleterious mutations. Therefore, we quantified individual plaque sizes for viruses isolated from vehicle-treated vs MOV-treated animals (Fig 2F). The average plaque size for virus exposed *in vivo* to MOV was significantly smaller than the average plaque size of virus not exposed to MOV. Thus, replication *in vivo* in the presence of MOV resulted not only in reduced virus titer, but also reduced fitness (i.e. small plaque variants) of the residual virus that retained infectivity.

### NHC antiviral activity correlates with mutation density within the LACV genome

NHC functions as a mutagenic ribonucleoside analog with the ability to reversibly base pair as either uridine (U) or cytidine (C) due to tautomerization. To examine this mechanism of action, we sequenced LACV genomes after replication in Vero cells. We used the Primer ID adaptation of a unique molecular identifier (multiplexed to cover several regions of the LACV genome—MPID). The use of Primer ID error correction of each sequenced viral genome provided accuracy to detect mutation density above the background error rate of 1 in 10,000 nucleotides sequenced [33–35]. Using this approach, we were able to establish a correlation between reduced virus titer and mutation density for virus replication in the presence of NHC (S1A Fig). Using the LACV cell culture model, a dose-dependent relationship was observed between the mutation rate of LACV and the concentration of NHC, with similar findings in Vero, hNSCs, and N2a cells (S1B Fig). Additionally, when looking at the mutation profile of the virus cultured with NHC, we saw the greatest increases in C-to-U and G-to-A transition mutation rates, which is consistent with our previous observation on the effect of NHC on the infectivity of multiple coronaviruses [21,23]. When we examined the mutation density for LACV grown in Vero cells in the presence of RBV or FAV, we did not see a significant increase in mutation rates except when the highest concentrations of RBV (100 μM) or FAV (100 μM) were used, indicating that NHC is markedly more potent at inducing viral mutations (S1A Fig).

**Fig 3:**
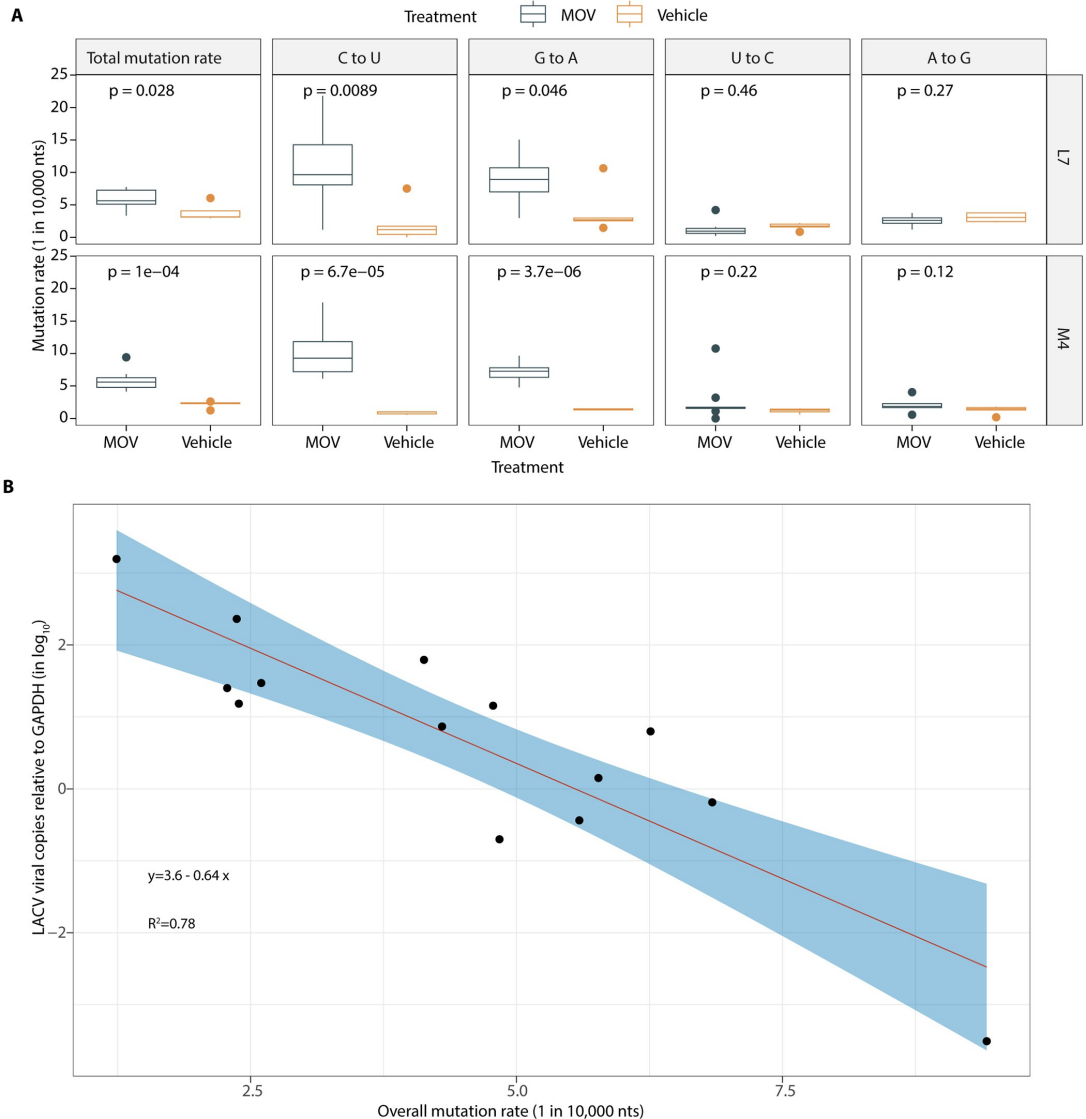
MPID-NGS of the mechanism of action of molnupiravir (MOV in LACV mouse model. (A) Mutation rate of LACV in the brain tissues after MOV (n = 9) or vehicle control (VC, n = 5) treatment. On the boxplots, the middle lines indicate the medians of the values, top and bottom parts of the boxes indicate the inter-quartile rage (IQR), the lines extending to the lowest and highest values indicate the range of 1.5*IQR, and the “*” symbols indicate the mean values of the mutation rate. Statistical comparisons were performed using Mann-Whitney U test. (B) Correlation of the overall mutation rate and LACV copy numbers in the brain tissues measured by qRT-PCR.

To confirm the mechanism of action of NHC *in vivo* was also through mutagenesis, we sequenced viral RNA in isolated brain tissue from mice with the onset of clinical signs or at 5 dpi that were infected with LACV and treated with MOV. In this model, the total mutation rate of the virus was increased by 2.8 per 10,000 bases in the presence of MOV when compared to vehicle controls (with the mutation rate averaged across the different genomic regions/amplicons sequenced). Specifically, the C-to-U transition rate was increased by 9.1 per 10,000 bases and the G-to-A transition rate was increased by 5.2 per 10,000 bases (Fig 3A), consistent with our findings with the LACV cell culture model. When we plotted the overall mutation rate of LACV exposed to MOV and LACV copy numbers in the murine brain tissues measured by qRT-PCR, we found a negative logarithmic relationship, which supports the lethal mutagenesis mechanism of MOV *in vivo* (Fig 3B). We estimate that each 1 mutation increase per 10,000 nucleotides resulted in an approximately 4.4-fold decrease in viral load in brain tissue.

### Antiviral effect of molnupiravir (MOV) given orally during IN inoculation of LACV

**Fig 4:**
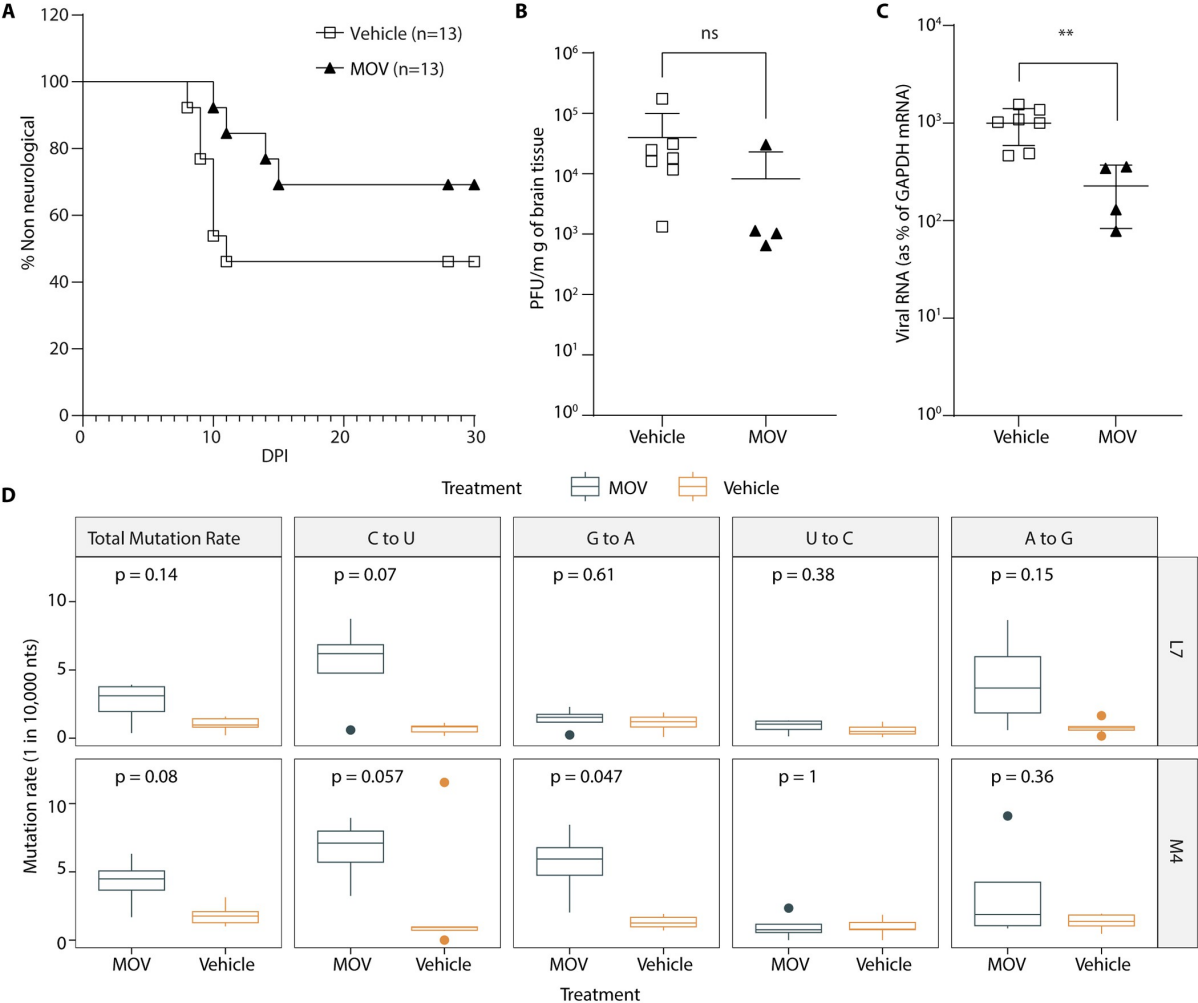
Molnupiravir (MOV) treatment increases survival after infection via an IN route. (A-C) A mix of both male and female C57BL/6 mice at the age of day 23 to 25 were infected with LACV (1x10^2^ pfu/mouse) through an IN route. Mice were treated orally with MOV (300 mg/kg, n = 13) or DMSO (n = 13) twice daily until the onset of clinical signs or 10 dpi, starting at day 0 (2 hrs prior to infection) and followed for development of clinical disease. Kaplar-Meier analysis was used to calculate the difference of clinical disease and statistical analysis (A). Brain tissue was harvested after animals showed clinical signs of the disease from the experiments (A). Out of 13 mice, 7 mice showed clinical signs in vehicle treated group, whereas 4 mice were clinical in MOV treated group. To quantify the virus in brain tissue we used (B) a plaque assay for quantifying infectious virus titer and (C) qRT-PCR for viral RNA (n = 7 for vehicle control and n = 4 for MOV treatment). Lines and error bars for each set indicate mean ± S.D. (D) Mutation density of LACV in brain tissue in presence (n = 4) or absence (vehicle, n = 7) of MOV treatment was measured by MPID-NGS in two sequenced regions (L7 and M4). On the boxplots, the middle lines indicate the medians of the values, top and bottom parts of the boxes indicate the inter-quartile rage (IQR), the lines extending to the lowest and highest values indicate the range of 1.5*IQR, and the “*” symbols indicate the mean values of the mutation rate. Statistical comparisons in panel (B-D) were performed using Mann-Whitney U test.

We have previously shown that infection with LACV induces vascular leakage which promotes viral neuroinvasion [42]. It is known that MOV (or NHC after release of the 5’-isopropyl ester group in the blood) has the ability to cross the blood-brain barrier (BBB) [43], however it is not known if MOV treatment attenuates neurological disease by mutating virus in the periphery prior to viral entry through the BBB or if MOV treatment would generate sufficient NHC in the brain to mutate virus and reduce disease of virus that rapidly moves to the CNS. To address this question and to further understand the ability of MOV to treat infections within the CNS, we performed an additional study where we infected mice with LACV (10^2^ pfu/ml) via an intranasal (IN) inoculation, which provides a direct route of access to the CNS through the olfactory nerve [42]. Mice were treated orally with MOV or vehicle control starting on the day of infection. LACV-infected mice treated with vehicle control had a clinical disease rate of about 45% (Fig 4A). MOV treatment decreased the incidence of neurological disease to about 23%, or about a 50% reduction in disease in this less pathogenic model (Fig 4A). Infectious virus titer in the brain was slightly, but not significantly decreased with some variability between mice (Fig 4B). However, viral RNA in the brain (Fig 4C) was significantly decreased in MOV-treated mice. When we sequenced the LACV RNA from the brain samples using MPID-NGS, we found increases in the same types of mutations as seen in the IP-inoculated mice, although the levels were reduced by about two-fold (Fig 4D). MOV increased both C-to-U mutations and G-to-A mutations compared to vehicle control, with C-to-U and G-to-A mutations increasing by 4.3 per 10,000 bases (Fig 4D). While these trends are the same as what was seen with virus sequenced after treatment in the IP infection model (Fig 3), the two-fold difference in mutation density in the treatment group reduced the number of comparisons that reached statistical significance (Fig 4D).

### Serial passage of LACV in presence of NHC to select for drug resistance virus

**Fig 5:**
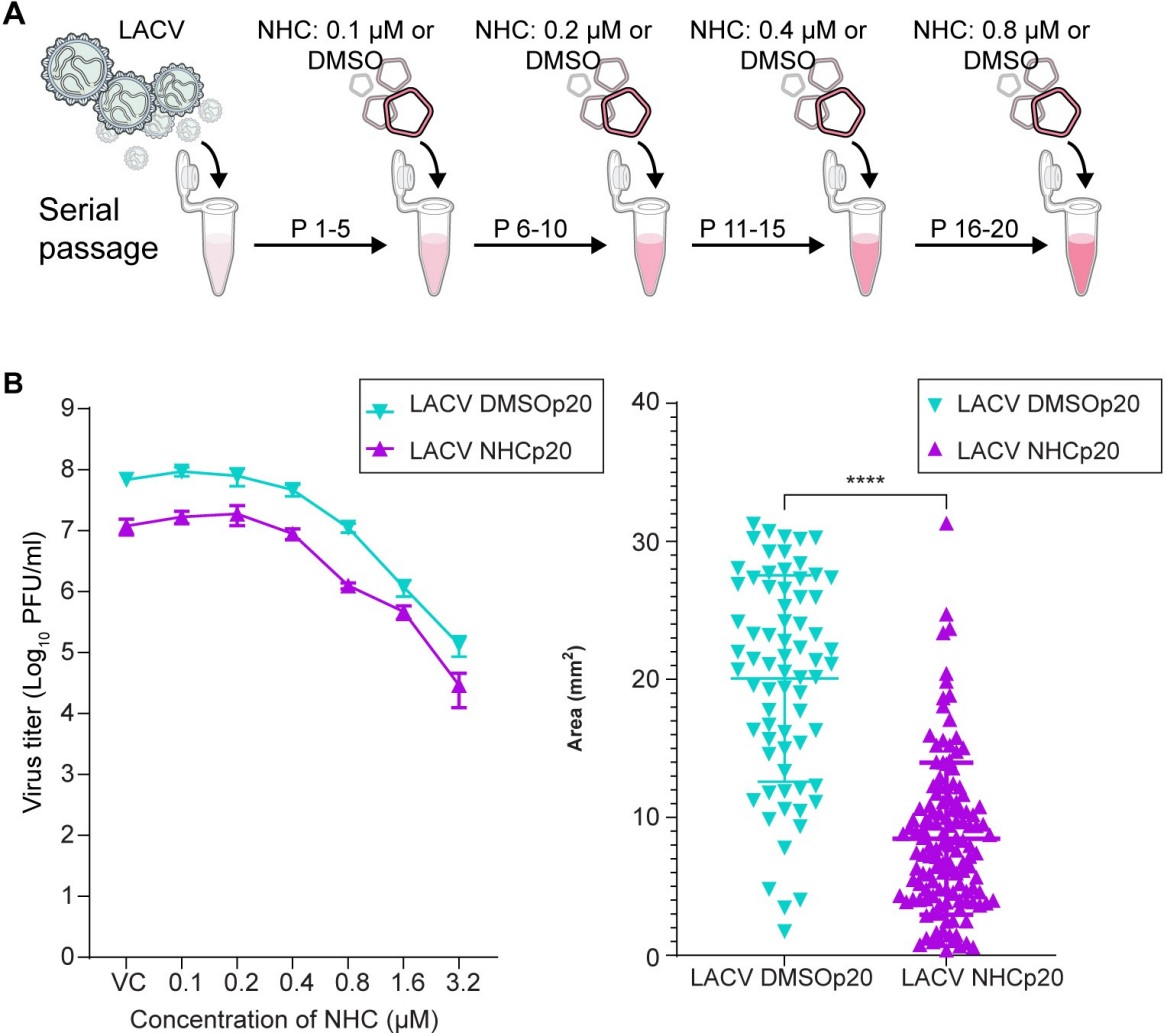
Serial passage of LACV in the presence of increasing NHC concentrations to select for resistance. (A) Study protocol of serial passaging of LACV in presence of DMSO or increasing concentrations of NHC (0.1 to 0.8 μM), with virus being passaged five times at each concentration. (B) Dose-dependent antiviral activity of NHC tested against the serially passaged LACV. Passaged viruses were used to infect Vero cells treated with different concentrations of NHC or with DMSO (VC). At 24 hr post infection (hpi), virus titer was measured from cell supernatant using a plaque assay. Each line graph represents the mean number of infectious viruses present in three individual wells of an independent experiment with an error bar indicating ± s.d. (C) After 20 passages the virus pools from passage with (n = 207) or without (n = 122) NHC were used to form plaques on Vero cells. Plaque sizes were imaged and their sizes measured; the distributions of plaque sizes for each virus group with the size distributions significantly different assessed as in Fig 2.

LACV was passaged in Vero cells in the presence of DMSO vehicle carrier (control) or with increasing concentrations of NHC from 0.1 to 0.8 μM (Fig 5A). After 20 passages (five at each of the increasing NHC concentrations), cell supernatants were harvested. The antiviral activity of NHC was determined in Vero cells for both the control virus and the NHC-selected virus. The EC_50_ values were the same (0.5 μM and 0.49 μM) for both the control and selected viruses (Fig 5B) and similar to the low passage parent virus (Fig 1D). Thus, serial passage of LACV in the presence of increasing concentrations of NHC failed to select for virus with reduced sensitivity to NHC. In this experiment the virus was not titered between each passage nor the titer normalized between the two viruses. This likely results in more replication of the control virus given it was not inhibited. However, this point just emphasizes that passage alone does not select for resistance to NHC, and in this protocol, passage in the presence of NHC did not select for resistance either.

While the goal of the passaging experiment was to try to select for resistance, given that the selective pressure is the incorporation of mutations we would again expect the NHC-exposed virus to be impacted by exposure to the drug. The inhibitory effect of NHC can be inferred in that the supernatant virus titer at the end of the selection period was approximately 15-fold lower than for the control passaged virus. To link this lower titer to a loss of fitness we again measured plaque size in the two passage 20 virus stocks. As can be seen in Fig 5C, the average plaque size for the virus population passaged in the presence of NHC is significantly smaller than the plaque size in the DMSO-treated control.

### Effect of NHC on other orthobunyaviruses

**Fig 6:**
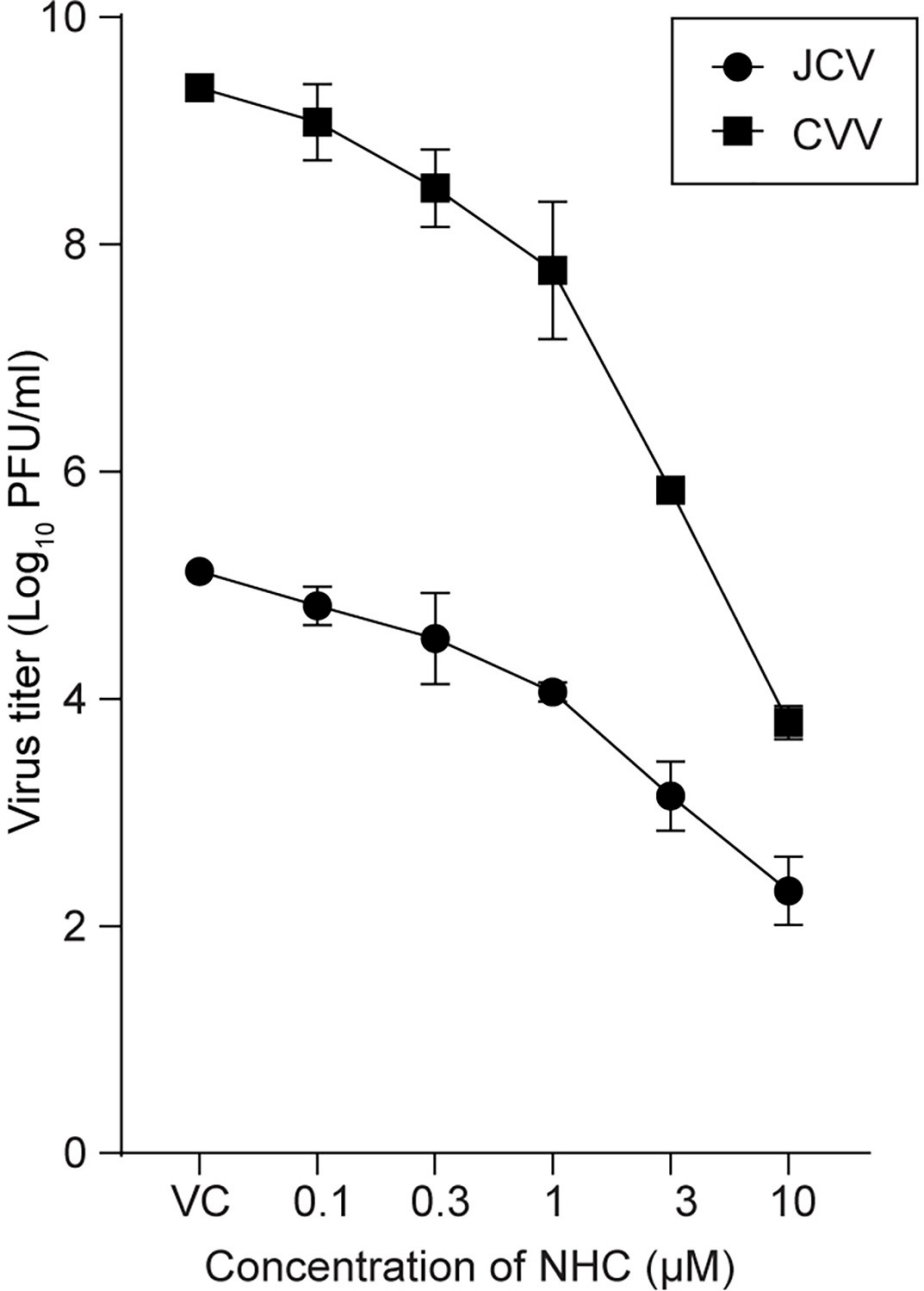
Treatment with NHC inhibits the replication of other orthobunyaviruses (JCV and CVV). Virus-infected cells were treated with different concentrations of NHC or DMSO (VC). At 24 hr post infection (hpi), virus titer was measured in the cell supernatant by plaque assay. Line graph represents the mean virus titer present in individual wells combined from three independent experiments with an error bar indicating ± s.d.

We also evaluated the ability of NHC to inhibit the replication of two other orthobunyaviruses, Cache Valley virus (CVV) and Jamestown Canyon (JCV). JCV belongs to the same serogroup as LACV but causes neurological disease in both children and adults. CVV is a member of the Bunyamwera serogroup and is the most common orthobunyavirus in the United States. Infections were done following the same protocol as for Fig 1. NHC treatment efficiently reduced CVV and JCV infectious virus production in Vero cells with EC_50_ values of 0.25 and 0.3 μM respectively (Fig 6), similar to LACV (Fig 1D). Thus, NHC inhibited virus replication for multiple orthobunyaviruses in different serogroups with similar potency.

## Discussion

In this study, we used LACV, a member of the California Serogroup (CSG) of orthobunyaviruses, as a representative neurotropic virus to study the antiviral effect of the mutagenic nucleoside NHC/MOV in a viral encephalitis model. After a comparative analysis of three mutagenic nucleoside analogs against LACV replication in different neuronal cell lines, we found that NHC was significantly more potent than RBV and FAV at inhibiting virus replication. We also showed *in vivo* efficacy of NHC, in that treatment with the prodrug MOV increased survival of LACV-infected mice and induced less fit and mutated virus in brain tissue. Although we did not measure uptake or intracellular states of these nucleoside/base precursors, it seems likely that differences in potency of the different analogs represent differences in uptake, initial metabolism to the 5’-triphosphate, and/or incorporation by the viral polymerase.

Suitable mouse models are essential for validating antiviral activity. In this study, we used a weanling C57Bl/6 mouse model which mimics LACV disease in humans. Weanling C57Bl/6 mice are susceptible to LACV infection either through IP (peripheral) or IN (direct access to the brain) route of inoculation. We initially used the IP route of inoculation which initiates a peripheral infection followed by neuroinvasion. MOV treatment significantly increased survival of LACV-infected mice and generated mutated and less fit virus in the brain (Fig 2). In a second experiment, we utilized the IN route of inoculation to determine if the drug when given orally was able to display an antiviral effect on virus replication in the brain. In this second experiment, there was a trend of increased survival with MOV treatment which was consistent with our previous IP inoculated model. Even in this stringent viral encephalitis model, we were still able to detect increased mutagenesis of the viral genome in the brain (Fig 4D) suggesting the presence of the drug within the CNS even after oral administration, consistent with a previous study using a VEEV infection model that employed oral dosing of the NHC form of the analog [43]. The current dosing of MOV in humans is 800 mg twice per day for treatment of SARS-CoV-2. This corresponds to a dose of approximately 13 mg/kg for the human dose, with a mouse equivalent dose of about 164 mg/kg [44]. Thus, our dosing of 300 mg/kg in mice is approximately twice the equivalent dose used in humans for treating SARS-CoV-2. It is possible that intrathecal administration could provide a more efficacious dosing strategy in cases of viral encephalitis. Also, while we did not specifically assess the presence of virus after treatment, most of the animals that were alive at the end of the 10 day treatment survived to the end of the observation period (28–30 days post infection). This suggests that viral rebound does not make a significant contribution to disease after MOV treatment.

NHC has been demonstrated to be an active antiviral against a number of RNA viruses including in VEEV, SARS-CoV-2, MERS-CoV, Ebola virus, LACV (and other California serogroup orthobunyaviruses), and others [18–21]. It is proposed that the mutagenic effect of MOV/NHC is due to the presence of the 4-oxime group, which can undergo tautomerization between amino and imino forms to allow MOV/NHC to ambiguously base pair with either adenosine or guanosine depending on its tautomeric form [45,46]. Since RNA viruses must undergo both plus strand and minus strand RNA synthesis where NHC can be incorporated, both G-to-A or C-to-U mutations appear in the plus/coding strand. These findings are consistent with the mechanism of action for MOV/NHC in LACV being through lethal mutagenesis, whereby increasing the mutation rate of the virus past its error threshold results in decreases in the viral population mean fitness and eventual viral population collapse due to the accumulation of deleterious mutations [47]. This is further supported by our findings in Fig 3B, where we found a negative logarithmic relationship between the overall mutation rate of LACV exposed to MOV and LACV copy numbers in the murine brain tissues measured by qRT-PCR. While lethal mutagenesis is the end point of excessive mutations, in practice the treatment likely is creating less fit virus and reduced virus titers that allow host immune mechanisms to contain the infection. The potency of NHC is likely due to its close similarity to cytidine, allowing it to be readily metabolized and incorporated. This small chemical difference from cytidine may also be the reason we were unable to select for resistance to NHC given the small chemical target required to distinguish between cytidine and NHC (Fig 5), although selection for low-level resistance to NHC has been reported for VEEV and murine hepatitis virus [20,48]. Resistance develops most easily when a substrate analog occupies a volume outside of the substrate envelope [49] which would support the weak resistance that has been selected against NHC due to its similarity with cytidine.

NHC meets the goal of being a broadly acting antiviral against RNA viruses, showing potency against a wide range of viruses, although the potential risk of host DNA genotoxicity will not be known for years [50]. Other available classes of antivirals tend to be very specific such that a specific identification must be made before they are used. Targeting a host function (e.g. cellular receptor, acidification of endosome, avoiding binding a host protein during release) will work against the viruses that share that specific feature. Chain terminating nucleoside analogs will work against any virus where the polymerase is able to incorporate them (and not excise them), although there is a need for a certain potency as the achievable level of nucleoside analogs *in vivo* has limitations. However, targeting of viral enzymes beyond the viral polymerase (with chain terminating nucleosides) typically makes inhibitors that are very specific to a group of closely related viruses, such as protease inhibitors to HIV-1 or SARS-CoV-2, or in the case of the influenza virus cap-dependent endonuclease inhibitor. As new viruses appear, the available drugs are quickly tested but often are not effective or show modest activity at high concentrations (which calls into question the mechanism of action). Thus the novelty of NHC as a potent mutagenic nucleoside that targets the genetic viability of RNA viruses broadly makes it conceptually different from other classes of antivirals.

Ribavirin has been previously studied against LACV, including in phase I, IIa, and IIb clinical trials in pediatric patients with severe LACV-E, however its efficacy remains unclear. While ribavirin has previously shown antiviral activity against LACV in cell culture, its efficacy in clinical trials was poor due to reduced penetration into the CNS, resulting in subtherapeutic CSF concentrations when ribavirin was administered *via* IV [17,51]. Additionally, when higher doses of ribavirin were given to increase CSF concentrations, not only did the levels of ribavirin still fail to consistently reach therapeutic levels, but the higher doses were associated with higher rates of adverse events, including hyperammonemia and pancreatitis, thus suggesting a narrow therapeutic window for ribavirin in the treatment of LACV-E [17]. Off-target effects (beyond mutagenicity) on the host have been suggested to contribute to toxicity associated with RBV treatment and may also serve as additional mechanisms of action for its modest observed antiviral effects [17]. Though RBV (and FAV) potentially act through several different mechanisms of action, including lethal mutagenesis, the weak potency of these drugs as viral mutagens compared to NHC may explain why NHC is a more potent antiviral, with this potency allowing it to show some antiviral efficacy *in vivo* at achievable levels of dosing [17,52].

*N*^4^-hydroxycytidine was first described as the mutagenic product of treating DNA with hydroxylamine [53] which highlights the genotoxicity of a mutagenic deoxyribonucleoside analog. However, given that deoxyribonucleotide precursors are synthesized from ribonucleoside diphosphates, this suggests that exposure of cells to a potent mutagenic ribonucleoside analog will also result in the incorporation into host DNA, and this mutagenic potential for NHC has been demonstrated in two experimental systems [50,54]. This potential genotoxicity risk of molnupiravir is of unknown magnitude on a population basis. Thus identifying settings where the risk/benefit ratio is strongly advantageous, such as infections where there is a high rate of morbidity, should be a focus for extending the use of mutagenic ribonucleosides.

## Conclusion

Our results highlight the potential of using mutagenic compounds, particularly NHC/MOV, in the treatment of CNS infections caused by RNA viruses, an observation that has also been made for the alphavirus VEEV [43]. Additionally, further study is needed to examine intrathecal administration of NHC for treatment of an encephalitic RNA virus infection to enhance the levels of drug concentrations within the CNS through direct introduction into CSF. The intrathecal delivery of NHC offers the potential to mitigate systemic drug toxicity-related adverse effects. This approach could potentially minimize the exposure of high-turnover cells at greatest risk of genotoxicity due to mutagenesis of DNA by the deoxyribonucleotide metabolite of NHC.

## Supporting information

S1 Fig Mechanism of action of NHC, RBV and FAV in cell models. (A) Substitution rate measured by MPID-NGS in the LACV Vero cell model. (B) Substitution rate measured by MPID-NGS in the LACV Na2 and hNSCs models.
(PDF)

S1 Table Cytotoxicity of three nucleoside analogs using the MTT assay. (DOCX)

S2 Table LACV MPID-NGS library prep primers. (DOCX)
