# Supplementary material for: N4-Hydroxycytidine/molnupiravir inhibits RNA virus-induced encephalitis by producing less fit mutated viruses
Source: PLoS Pathog. 2024 Sep 30;20(9):e1012574. doi: 10.1371/journal.ppat.1012574 (PMC11493283; doi:10.1371/journal.ppat.1012574)
Supplement: S1 Fig — (A) Substitution rate measured by MPID-NGS in the LACV Vero cell model. (B) Substitution rate measured by MPID-NGS in the LACV Na2 and hNSCs models. (PDF) [file ppat.1012574.s001.pdf]

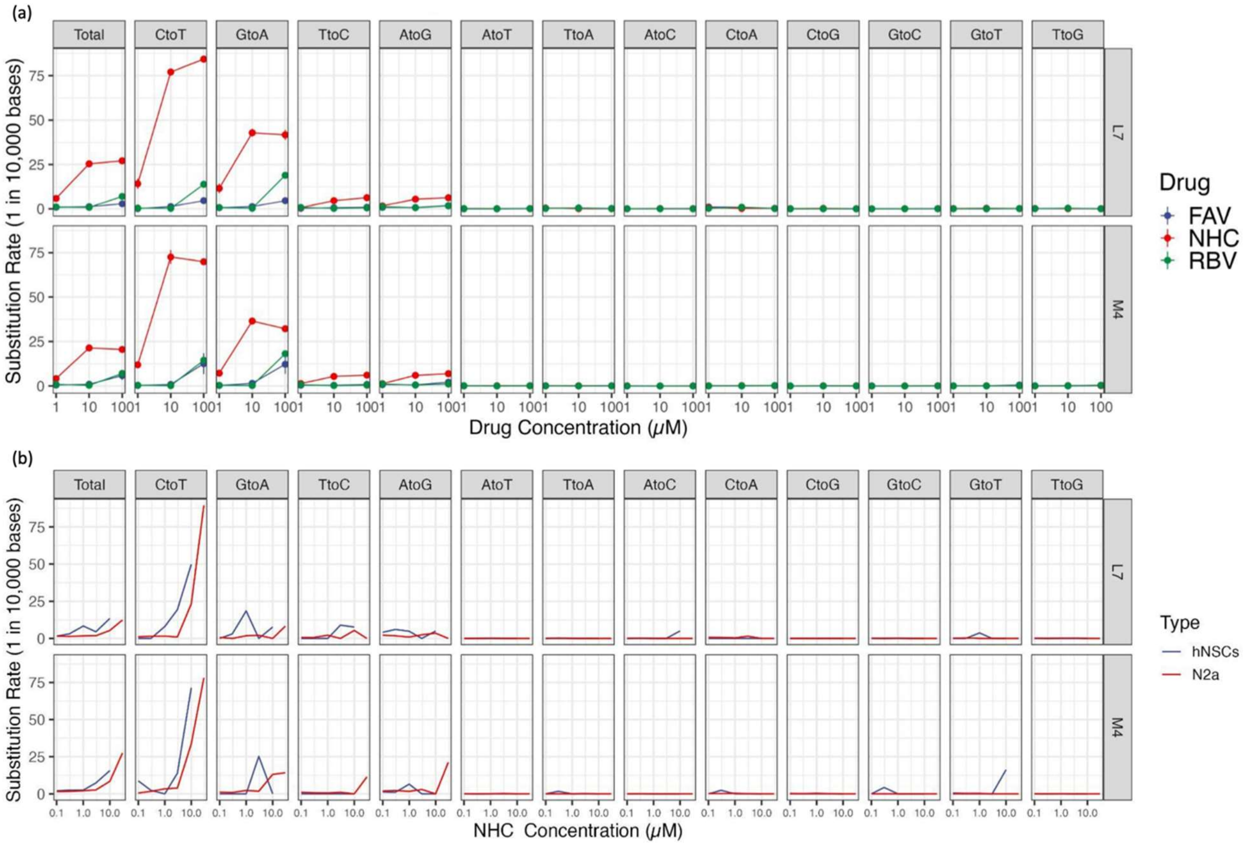

Supplemental Fig. 1. Mechanism of action of NHC, RBV and FAV in cell models. (A) Substitution rate measured by MPID-NGS in the LACV Vero cell model. (B) Substitution rate measured by MPID-NGS in the LACV Na2 and hNSCs models.
