# Supplementary material for: N4-Hydroxycytidine/molnupiravir inhibits RNA virus-induced encephalitis by producing less fit mutated viruses
Source: PLoS Pathog. 2024 Sep 30;20(9):e1012574. doi: 10.1371/journal.ppat.1012574 (PMC11493283; doi:10.1371/journal.ppat.1012574)
Supplement: S1 Table — (DOCX) [file ppat.1012574.s002.docx]

**Supporting Information**

**S1 Table.** Cytotoxicity of three nucleoside analogs by MTT assay.

| Drugs | CC_50_ (μM) | |
| --- | --- | --- |
|  | Vero cells | hNSCs |
| RBV | 1777.5 | 1264.7 |
| FAV | 3341.2* | 1059.8* |
| NHC | 203.1 | 131.9 |

*CC_50_ of FAV was copied and converted to μM from μg/ml and represented here.

**Method**: Cells were seeded in 96-well plates (1-2x10^4^ cells/well) after attachment different concentrations of each drug containing fresh media were added to each well, in triplicate. DMSO was used as a control. At 24h post-treatment, MTT assays were carried out according to the manufacturer’s instructions (Invitrogen, ThermoFisher Scientific). The absorbance was measured using the Synergy 4 cell plate reader (BioTek) at 540 nm. This assay was used to estimate the drug-induced cytotoxicity using the following formula: (sample absorbance/DMSO absorbance) × 100%. The CC50 (50% cytotoxic concentration) was determined by extrapolating the dose–response curve.
