# Supplementary material for: N4-Hydroxycytidine/molnupiravir inhibits RNA virus-induced encephalitis by producing less fit mutated viruses
Source: PLoS Pathog. 2024 Sep 30;20(9):e1012574. doi: 10.1371/journal.ppat.1012574 (PMC11493283; doi:10.1371/journal.ppat.1012574)
Supplement: S2 Table — (DOCX) [file ppat.1012574.s003.docx]

**Supporting Information**

**S2 Table.** LACV MPID-NGS library prep primers.

| L_PID11 | GTGACTGGAGTTCAGACGTGTGCTCTTCCGATCTNNNNNNNNNNNCAGTTCCTGTGGGTAGAGGATAGG | cDNA primer. Targeting LACV RdRp gene found on the large genomic segment. |
| --- | --- | --- |
| M_PID11 | GTGACTGGAGTTCAGACGTGTGCTCTTCCGATCTNNNNNNNNNNNCAGTAATCCGATAGATGTCCCAGC | cDNA primer. Targeting LACV medium genomic segment. |
| L_AD | GCCTCCCTCGCGCCATCAGAGATGTGTATAAGAGACAGNNNNAAGGCCAGAAAACGTCAAAG | 1^st^ round PCR forward primer. Targeting LACV RdRp gene found on the large genomic segment. |
| M_AD | GCCTCCCTCGCGCCATCAGAGATGTGTATAAGAGACAGNNNNTCCTGACGTAAAGCTCATCC | 1^st^ round PCR forward primer. Targeting LACV medium genomic segment. |
